# Potential Effects of Low-Level Toluene Exposure on the Nervous System of Mothers and Infants

**DOI:** 10.3390/ijms25116215

**Published:** 2024-06-05

**Authors:** So Yeon Yu, Seung Hwan Kim, Jeong Hyeop Choo, Sehun Jang, Jihyun Kim, Kangmo Ahn, Seung Yong Hwang

**Affiliations:** 1 Institute of Natural Science & Technology, Hanyang University ERICA, 55 Hanyangdaehak-ro, Sangnok-gu, Ansan 15588, Republic of Korea; yusso3027@naver.com; 2 Department of Bio-Nanotechnology, Hanyang University, 55 Hanyangdaehak-ro, Sangnok-gu, Ansan 15588, Republic of Korea; kandoli1@daum.net; 3 Department of Molecular & Life Science, Hanyang University, 55 Hanyangdaehak-ro, Sangnok-gu, Ansan 15588, Republic of Korea; cnwjdguq@naver.com; 4 Department of Pediatrics, Samsung Medical Center, Sungkyunkwan University School of Medicine, Seoul 06351, Republic of Korea; unitysky1@gmail.com (S.J.);; 5 Department of Health Sciences and Technology, Samsung Advanced Institute for Health Sciences & Technology, Seoul 06355, Republic of Korea; 6 Department of Medicinal and Life Sciences, Hanyang University ERICA, 55 Hanyangdaehak-ro, Sangnok-gu, Ansan 15588, Republic of Korea; 7 Department of Applied Artificial Intelligence, Hanyang University ERICA, 55 Hanyangdaehak-ro, Sangnok-gu, Ansan 15588, Republic of Korea

**Keywords:** toluene, maternal exposure, nervous system, GREEN cohort, epigenetics, mRNA expression

## Abstract

In day-to-day living, individuals are exposed to various environmentally hazardous substances that have been associated with diverse diseases. Exposure to air pollutants can occur during breathing, posing a considerable risk to those with environmental health vulnerabilities. Among vulnerable individuals, maternal exposure can negatively impact the mother and child in utero. The developing fetus is particularly vulnerable to environmentally hazardous substances, with potentially greater implications. Among air pollutants, toluene is neurotoxic, and its effects have been widely explored. However, the impact of low-level toluene exposure in daily life remains unclear. Herein, we evaluated 194 mothers and infants from the Growing children’s health and Evaluation of Environment (GREEN) cohort to determine the possible effects of early-life toluene exposure on the nervous system. Using Omics experiments, the effects of toluene were confirmed based on epigenetic changes and altered mRNA expression. Various epigenetic changes were identified, with upregulated expression potentially contributing to diseases such as glioblastoma and Alzheimer’s, and downregulated expression being associated with structural neuronal abnormalities. These findings were detected in both maternal and infant groups, suggesting that maternal exposure to environmental hazardous substances can negatively impact the fetus. Our findings will facilitate the establishment of environmental health policies, including the management of environmentally hazardous substances for vulnerable groups.

## 1. Introduction

The severity of environmental changes and pollution is a growing challenge, owing to changes in lifestyle and technological development [1]. Despite ongoing efforts to manage and improve environmental changes at the national level, exposure to environmentally hazardous substances can easily occur through breathing. Furthermore, environmentally hazardous substances have been identified in indoor spaces where individuals stay and live for prolonged periods of time [2]. Among the various environmentally hazardous substances, toluene is a volatile organic compound that easily volatilizes into the air and is present in both indoor and outdoor spaces [3]. Toluene is naturally generated by the incomplete combustion of natural fuel sources, such as natural gas and petroleum or released by industries including paint, lacquer, adhesive, ink, and wood [4,5]. Upon release into the atmosphere, water bodies, and soil, toluene causes extensive environmental pollution through atmospheric dispersion and infiltration into soil and groundwater [6]. It is typically rapidly inhaled via respiration and slowly absorbed through skin and eye contact. Chronic toluene exposure can exert negative effects on the nervous system, including memory loss, difficulties in concentrating, and attention deficit [7]. Therefore, toluene is classified as a neurotoxic substance. One study reported that babies born to women working with organic solvents did not differ from those in the control group in terms of cognition, language, and motor skills; however, children exposed to organic solvents at the fetal stage scored low in the neurobehavioral sub-test. Accordingly, maternal exposure to hazardous substances can affect the fetus and lead to a subtle deterioration of function [8]. Additionally, toluene exposure during pregnancy can contribute to the development of hydranencephaly [9]. Exposure to environmentally hazardous substances during pregnancy can negatively impact both the mother and the fetus. Moreover, given that the fetus develops during pregnancy, it is particularly vulnerable to environmental pollutants, with substantially greater implications, and these effects can be lifelong [10,11]. Therefore, it is important to comprehensively clarify and confirm the possible effects of maternal exposure to hazardous substances.

The impact of exposure to environmental pollutants in early life on neurodevelopment and the nervous system is an area of growing interest [12]. Therefore, the effects of environmentally hazardous substances on the human body are being explored using various methods [13,14,15]. However, elucidating the relationship between environmental exposure and human health can be challenging. Therefore, it is crucial to uncover and verify biological responses and impact, along with biomonitoring of environmental exposures, using various omics technologies [16,17]. Omics technology can help understand the underlying cause and progression of a disease [18]. In particular, a close relationship exists between environmentally hazardous substances and epigenetic changes. Harmful environmental factors function as external factors capable of inducing epigenetic changes and regulating gene expression, resulting in disease onset or affecting disease prognosis [19]. Among these epigenetic changes, DNA methylation has been shown to impact various biological processes and is involved in disease development [20,21].

Accordingly, in the current study, we aimed to determine the effect of the level of toluene exposed during daily life on the nervous system of the mother and fetus using various omics technologies. Herein, we employed the small GREEN cohort recruited to study environmental diseases caused by environmentally hazardous substances. First, the level of toluene exposure in real life was determined, and potential effects of epigenetic changes and altered mRNA expression that may occur due to exposure were confirmed.

## 2. Results

### 2.1. Characteristics of Human Participants

Participant information and the results of the metabolic product analysis are shown in Table 1. Participants mostly lived in metropolitan areas, including 21 residents of Gyeonggi-do, 56 of Seoul, 1 of Incheon, 1 of Chungcheong-do, and 18 of missing value. The average age of the mothers was 37.1 years, and the infants were newborns. The majority of mothers had one to two childbirth experiences, and only a small number of participants had given birth to three or more children. Most mothers were non-smokers, with three smokers (4.2%) in the low-exposure group and one in the high-exposure group (4.0%). At birth, the height and weight of both the low- and high-exposure groups were found to be normal. Additionally, the head circumference of the high-exposure group was within the normal range, with a mean of 34.2 cm, showing no significant difference when compared to the low-exposure group [22]. Although the developmental test was not performed in all infants, when conducted at 6 and 12 months, it revealed that all assessed infants were normal.

### 2.2. Toluene Exposure

The urinary level of N-acetyl-S-(benzyl)-L-cysteine (BMA), a toluene metabolite, was measured to confirm toluene exposure levels among study participants (Table 2). Toluene exposure level was detected in all samples, with no missing values. However, the level of exposure was not high because exposure was measured during day-to-day living and in pregnant women, who are more cautious than general adults. 

The maternal toluene exposure level (geometric mean) was 3.73 μg/g creatinine, which was lower than the geometric average toluene exposure level of Korean adults in 2020 (5.02 μg/g creatinine) [23]. Although we assessed various factors that could affect toluene exposure, none showed statistical significance (*p* ≥ 0.05). Accordingly, we confirmed that residence, age, smoking, and number of births were not associated with toluene exposure (Table 3).

### 2.3. Toluene Exposure Can Negatively Affect the Nervous System by Inducing Epigenetic Changes

Toluene exposure induced changes in the differentially methylated region (DMR). Herein, we found that 102,195 (57,136 hyper-regulated and 45,059 hypo-regulated) regions in the maternal group and 659,770 (647,931 hyper-regulated and 11,839 hypo-regulated) regions in the infant group were significantly altered. Accordingly, methylation-regulated genes were identified. In the maternal group, 30 mRNAs were hyper-methylated and downregulated, and 39 mRNAs were hypo-methylated and upregulated. In the infant group, four mRNAs were hyper-methylated and downregulated, and eighteen mRNAs were hypo-methylated and upregulated. We confirmed that several of these play a role in nervous system-related functions or nervous system-related diseases (Table 4 and Table 5).

**Figure 1:**
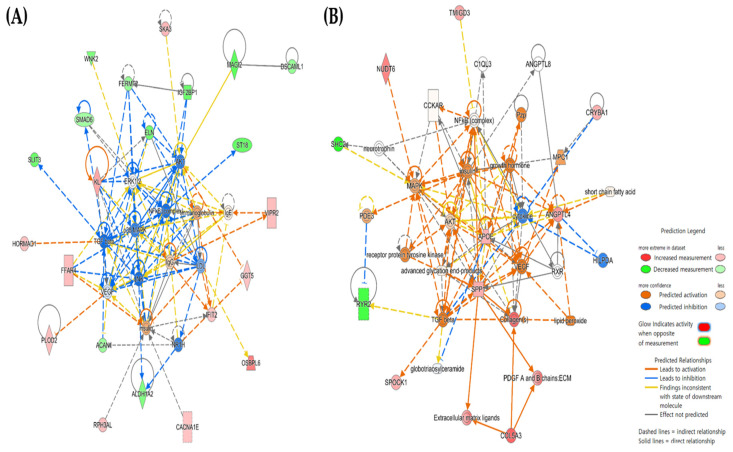
Gene interaction network map of each group. (**A**) Maternal group and (**B**) infant group. This network is composed of the top network, and was analyzed using the QIAGEN Ingenuity Pathway Analysis program.

Using QIAGEN Ingenuity Pathway Analysis (IPA), we investigated the interaction networks between mRNAs with methylation-induced significantly altered expression in each group (Figure 1). In the maternal group, genes related to cancer (such as glioma and neuroepithelial tumor) and organismal injury and abnormalities ranked high. Conversely, in the infant group, genes associated with lipid metabolism and molecular transport were prominently involved.

### 2.4. Toluene Exposure Leads to Hypermethylation-Induced ALDH1A2 Downregulation 

**Figure 2:**
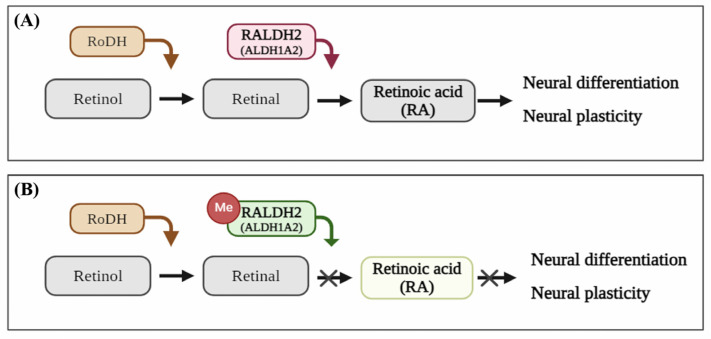
(**A**) Summary of retinoic acid (RA) signaling pathway. (**B**) Summary of RA signaling pathway following toluene exposure. In general, RA is metabolized by retinaldehyde dehydrogenase 2 (RALDH2) and plays a role in neuronal differentiation, development, and plasticity. However, toluene exposure downregulates RALDH2 (*ALDH1A2*) expression owing to methylation, which hinders RA metabolism and activation; this, in turn, may negatively impact neuronal differentiation, development, and plasticity. Figure created using BioRender (https://biorender.com/, accessed on 3 April 2024).

Among mRNAs whose expression was regulated by methylation due to maternal toluene exposure, we identified ALDH1A2, also called retinaldehyde dehydrogenases 2 (RALDH2), which plays a role in converting retinal to retinoic acid (RA) [56]. Hyper-methylation downregulates *ALDH1A2* mRNA expression, which disrupts RA synthesis. This can ultimately reduce RA levels, which can negatively impact the nervous system (Figure 2) [57,58]. Likewise, in the infant group, *ALDH1A2* mRNA showed a tendency toward downregulated expression (logFC −0.29, FDR 1), although was not statistically significant, owing to hyper-methylation (logFC 1.00, *p* 0.04).

### 2.5. Toluene Exposure Upregulates Genes Involved in Inflammatory Response 

The high-exposure group (top 25% and with higher exposure) exhibited significant changes in expression when compared with the low-exposure group; however, the difference in the expression between the two groups was non-significant. In the maternal group, 330 mRNAs and non-coding RNAs (ncRNA) were significantly altered (220 upregulated and 110 downregulated). In the infant group, 89 mRNAs and ncRNAs were significantly altered (39 upregulated and 50 downregulated). Comparing the maternal and infant groups, the expression patterns of seven upregulated mRNAs and ncRNAs and eight downregulated mRNAs and ncRNAs were common between the two groups. Among mRNAs with the same expression pattern in the maternal and infant groups, CXCL10 was overexpressed, confirming the suppression of neurogenesis or involvement in various neurodegenerative diseases [59]. In addition, upregulated mRNAs in each group were involved in inflammatory response at a high rate. Accordingly, a substantial number of genes were upregulated in neurodegenerative diseases and various neurological diseases (Table 6).

## 3. Discussion

During day-to-day living, humans are exposed to numerous environmentally hazardous substances that impact the human body. However, immediate and evident health effects upon exposure are extremely rare. Nevertheless, exposure to these substances exerts a distinctly negative impact on health [70]. Moreover, in the case of pregnant women, exposure can negatively impact both the mother and the fetus [10]. Therefore, in the current study, we focus on the effects of toluene levels commonly encountered in daily life on the nervous system. Simultaneously, we evaluated pregnant women to determine the possible effects on the mother and infants (fetus).

To assess the extent of toluene exposure in daily life, we evaluated urinary BMA levels. Urinary BMA is a valid indicator for toluene exposure assessment in National Biomonitoring Programs in various countries, including the Republic of Korea, the United States, and Canada [71,72,73,74]. The results of the urinary BMA measurements indicate that toluene (i.e., the toluene metabolite BMA) could be detected in trace amounts in all participants, with no missing data. The average toluene exposure level (geometric mean) was 3.73 μg/g creatinine for the maternal group; this value was lower than the average toluene levels reported for adults (5.02 μg/g creatinine) in the 2020 Republic of Korea National Environmental Health Basic Survey [23]. The toluene exposure level-related differences could be attributed to several factors. First, the National Environmental Health Survey includes adults aged at least 19 years, encompassing both males and females, potentially representing a wide range of living environments and occupational exposure levels. In contrast, the participants in this study included pregnant women, who might generally tend to be minimally exposed to toluene. Second, the National Environmental Health Survey contains data from various regions nationwide; however, this study was limited to a specific region (the metropolitan area), potentially leading to regional differences. Despite these factors contributing to the observed differences, it is evident that all participants in this study were exposed to either general or low toluene levels. These findings are also evident from the participants’ basic health information. They were able to give birth without any significant concerns during pregnancy, and the height, weight, and head circumference of their children were found to be normal (2017 average for newborns in Republic of Korea). Additionally, developmental assessments (Denver Development Screening Test II) at 6 and 12 months confirmed that their growth and development were progressing normally. Additionally, binomial logistic regression analysis was performed to determine whether the mother’s residence, age, smoking status, and number of births affected toluene exposure. Herein, we confirmed that the examined factors did not impact toluene exposure (with *p* ≥ 0.05).

After determining the maternal level of toluene exposure, the participants were divided into low- and high-exposure groups based on the top 25% of the group. For the infant group, the maternal group was applied with no changes, and omics data analysis was performed. Exposure levels of toluene encountered in daily life induced epigenetic changes and altered mRNA expression, resulting in the upregulation of 39 and 18 mRNAs via hypo-methylation, and the downregulation of 30 and 4 mRNAs owing to hyper-methylation in the mother and infant groups, respectively. Most upregulated mRNAs were identified in glioblastoma or Alzheimer’s disease [24,25,27,31,32,33], whereas downregulated mRNAs were found to reduce the size and density of axons, dendrites, and synapses or reduce synaptic connectivity [35,36,40,46,50,51]. Additionally, using QIAGEN IPA (v17.6) analysis, we analyzed interactions between mRNAs for each group, identifying the highest network (Figure 2).

In the maternal group, genes related to cancer (such as glioma and neuroepithelial tumor) and organismal injury and abnormalities ranked high. As observed in Table 4 and Table 5, upregulated mRNAs, including *SKA3*, *PLOD2*, *VIPR2*, and *GGT5*, are implicated in neuroblastoma. Conversely, downregulated mRNAs, such as *MAGI2*, *ST18*, *ALDH1A2*, *FERMT2*, and *DSCAML1*, appear to negatively impact neurites, axons, and synapses. In the infant group, the marked involvement of genes associated with lipid metabolism and molecular transport was identified, particularly their upregulated expression. For example, *APOE* and *SPP1* mRNA participate in lipid metabolism and are related to Alzheimer’s disease [75]. APOE reportedly plays an important role in maintaining brain lipid homeostasis and regulating microglial responses and related lipid metabolism during nerve injury, which may contribute to the development of neurodegenerative diseases [76,77]. In addition, SPP1, a disease-associated microglia gene, was confirmed to be upregulated in AD [78].

In particular, hypo-methylation downregulated *ALDH1A2* mRNA expression in the maternal group. ALDH1A2 (also called retinaldehyde dehydrogenases 2, RALDH2) irreversibly metabolizes RA [56]. In general, RA is a vitamin A metabolite that plays an important role in various areas such as vision, immune function, and reproduction. RA is involved in adult neurogenesis in the hippocampus, including neural plasticity, learning, and memory, and regenerative motor neuron axon growth. Furthermore, it contributes to the development of various systems and tissues during embryogenesis [56,57]. However, the downregulated ALDH1A2 (RALDH2) expression hinders RA metabolism; consequently, RA fails to function accurately if the signal is not activated. In adults, disrupted or reduced RA signaling may result in motor neuron degeneration and degenerative neurological diseases such as Alzheimer’s disease. Additionally, a decrease in RA during development can cause developmental abnormalities and neurobehavioral abnormalities in the fetus [57,58]. In the infant group, *ALDH1A2* mRNA showed a tendency to be under-regulated (logFC −0.29, FDR 1), although this effect was not statistically significant owing to hyper-methylation (logFC 1.00, *p* 0.04). Moreover, one study confirmed that *Raldh2* overexpression could improve ethanol-induced developmental defects [79]. Consistently, children exposed to organic solvents during the prenatal period failed to obtain good results in neurobehavioral subtests, and these results can be viewed as supporting evidence for our findings [8]. Therefore, ALDH1A2 may be a biomarker for nervous system-related diseases due to toluene exposure.

Although expression was not regulated by methylation, it was confirmed that many of the significantly over-expressed mRNAs were involved in the inflammatory response (GO:0006954). These mRNAs are typically involved in protecting the brain from inflammatory reactions in the cranial nerves caused by harmful substances or infections. However, chronic inflammatory responses activate glial cells and promote cytokine secretion, resulting in nerve damage [80], neurodegeneration caused by this chronic neuroinflammatory response is typically observed in various neurodegenerative diseases. *IL6* (logFC 2.64, FDR 7.37 × 10^−24^) mRNA has the highest expression among genes involved in various inflammatory responses. Interleukin (IL)-6 is a pro-inflammatory cytokine with various actions, and plays a role in maintaining homeostasis in the nervous system. However, excessive production or elevated levels of IL-6 can lead to neuroinflammatory reactions and neurodegeneration (which are known to be related to cognitive impairment) [60,81]. The levels of tumor necrosis factor-α and IL-β (including IL6) are significantly increased in Parkinson’s disease, a neurodegenerative disease [82]. In addition, *CXCL10* mRNA, which was upregulated in both maternal and infant groups, is known to be highly upregulated in the central nervous system when a neuroinflammatory response occurs [83]. In particular, one study reported that the secretion of pro-inflammatory cytokines increases with increasing exposure to benzene, toluene, ethylbenzene, and xylene (BTEX) compounds, which can affect inflammation [84]. These results indicate that the level of toluene exposure in daily life can contribute to neuroinflammatory responses.

## 4. Materials and Methods

### 4.1. Human Participants and Ethical Approval

This study used the small (GREEN) cohort that recruited 151 mothers and infants from 2017–2021 to study the environmental diseases caused by environmentally hazardous substances. Whole blood and cord blood were obtained from 97 matched mother-infant pairs, and peripheral blood mononuclear cells (PBMCs) were isolated. PBMCs were obtained using density gradient centrifugation with 6% Hetastarch solution (STEMCELL Technologies, Vancouver, BC, Canada) and Ficoll-Paque PLUS (GE Healthcare Life Sciences, Marlborough, MA, USA). Maternal urine was also collected. gDNA and total RNA were obtained from PBMCs using the Invitrogen PureLink Genomic DNA Mini Kit (Thermo Fisher Scientific, Waltham, MA, USA) and RNAiso Plus reagent (Takara Bio, San Jose, CA, USA) according to the manufacturer’s recommended method. In addition, the maternal urinary BMA concentration was measured using liquid chromatography-mass spectrometry performed by Smartive Co., Ltd (Hanam, Republic of Korea). The Korean version of the Denver Development Screening Test II was administered to confirm developmental delays when the infant was 6 and 12 months old. In total, four items (personal-social development, fine motor and adaptive development, language development, and motor development) were evaluated by experts using the test tools. This study was approved by the Institutional Review Board (IRB #2016-12-111) of Samsung Medical Center, and written informed consent was obtained from the parents participating in this study.

### 4.2. Selection of Toluene-Exposed Groups

The maternal participants in the top 25% and with higher exposure (5.99 μg/g creatinine) were assigned to the high-exposure group, whereas those in the bottom 75% exposure were assigned to the low-exposure group. Considering the infant group, the infants of mothers assigned to the high-exposure group were set as the high-exposure group. 

### 4.3. Methylated DNA Immunoprecipitation Sequencing (MeDIP) Sequencing and DMR Analysis

In brief, 500 ng of gDNA obtained from PBMCs was fragmented to an appropriate size (100–200 bp) using a Bioruptor pico sonicator (Diagenode, Denville, NJ, USA). MeDIP DNA was obtained via immunoprecipitation. Subsequently, the end-repairing and adenylated 3′ ends processes were performed using TruSeq ChIP Library Preparation Kit (Illumina, San Diego, CA, USA) in accordance with the according to the manufacturer’s guidelines, and DNA bound to the adapter index was amplified to create a library. The size of the constructed library was confirmed, quantified using qPCR, and sequenced using the Nextseq500 sequencing system (Illumina) with a read length of 2 × 75 bp. Preprocessing was performed using Trimmomatic (v0.40), bowtie2 (v2.4.2), SAMtools (v1.11), and Picard (v2.25.0) programs utilizing the FASTQ file obtained through sequencing. At this time, three pairs were excluded from the subsequent analysis owing to quality issues. The MEDIPS package in R (v4.2.1) was used for DMR analysis, and regions with a significant level (|fold change (FC)| > 1.5 and *p*-value < 0.05) were selected. 

### 4.4. mRNA Sequencing and Differentially Expressed Gene (DEG) Analysis

Ribosomal RNA was extracted from 100 ng of total RNA obtained from PBMCs using the TruSeq Stranded Total RNA Sample Prep Kit with Ribo-zero Globin (Illumina) according to the manufacturer’s guidelines. A cDNA library was created, and its size was checked and quantified using quantitative polymerase chain reaction (qPCR). This library was sequenced using a NovaSeq 6000 sequencing system (Illumina) at 2 × 100 bp read length. Preprocessing was performed using Trimmatic (v0.40) and STAR (v2.7.8a) programs using FASTQ files obtained through sequencing. Data were normalized using the estimateCommonDisp method in the edgeR package of R (v4.2.1), and expression changes at a significant level (|FC| > 1.5 and false discovery rate (FDR) < 0.05) were selected. Additionally, DEG and DMR results were integrated and analyzed to identify genes whose expression was altered due to epigenetic changes.

### 4.5. Statistical and Functional Analysis

To determine the statistically significant impact of toluene exposure on various factors, we performed binomial logistic regression analysis using IBM SPSS Statistics (v25; IBM Corp., Armonk, NY, USA). Additionally, the function and network analysis of selected genes with significantly regulated expression were performed using QIAGEN Ingenuity^®^ Pathway Analysis (QIAGEN IPA, v17.6, Qiagen Germantown, MD, USA) and DAVID Bioinformatics Resources 6.8.

## 5. Conclusions

This study investigates the potential effects of toluene exposure at levels commonly encountered in everyday situations on the nervous system of pregnant mothers and infants (fetus). Toluene exposure leads to DNA methylation, resulting in upregulated expression of several mRNAs associated with gliomas and Alzheimer’s disease. Conversely, downregulated mRNAs are linked to reductions in dendritic spine size, density, and synaptic connectivity. Remarkably, similar results were observed in indirectly exposed infants, mirroring those seen in directly exposed mothers. These findings highlight the potential negative impact of environmental hazards, including toluene exposure, on both maternal and fetal health. Consequently, this research provides a foundation for understanding and managing environmental risks faced by vulnerable populations, including pregnant women.

## Figures and Tables

**Table 1:** Information of study participants.

Personal Characteristics	Low Exposure	High Exposure	*p*
Maternal group	*n* = 72	*n* = 25	
BMA (μg/g creatinine)	2.9 ± 1.3	30.5 ± 96.0	0.163
Age	37.5 ± 3.0	36.8 ± 3.6	0.425
Smoking			0.368
No	58 (80.6%)	17 (68.0%)	
Yes	3 (4.2%)	1 (4.0%)	
Missing data	11 (15.3%)	7 (28.0%)	
Childbirth			0.724
1	32 (52.5%)	10 (55.6%)	
2	21 (34.4%)	7 (38.9%)	
3	5 (8.2%)	0 (0.0%)	
4	2 (3.3%)	1 (5.6%)	
5	1 (1.6%)	0 (0.0%)	
Infant group	*n* = 72	*n* = 25	
Sex			0.213
Male	43 (59.7%)	10 (40.0%)	
Female	21 (29.2%)	10 (40.0%)	
Missing data	8 (11.1%)	5 (20.0%)	
Height	50.0 ± 1.9	49.3 ± 2.0	0.232
Weight	3.3 ± 0.4	3.2 ± 0.4	0.871
Head circumference	34.5 ± 1.3	34.2 ± 1.3	0.460
Denver test (6 M)			0.076
Normal	62 (86.1%)	17 (68.0%)	
Untested	10 (13.9%)	8 (32.0%)	
Denver test (12 M)			0.035
Normal	62 (86.1%)	16 (64.0%)	
Untested	10 (13.9%)	9 (36.0%)	

Data are shown as mean ± standard deviation (SD). BMA, N-acetyl-S-(benzyl)-L-cysteine; 6 M, 6 months; 12 M, 12 months.

**Table 2:** Toluene metabolite (BMA) evaluation results in maternal group.

BMA (μg/g Creatinine)	Maternal Group	
	Low-Exposure	High-Exposure
Limit of Detection (LOD)	0.03 (μg/L)	
Minimum	0.33	5.99
Maximum	5.74	489.83
Geometric mean	2.52	11.47
Arithmetic mean	2.86	30.46
Standard Deviation (SD)	1.26	95.97

**Table 3:** Effects of various factors on toluene exposure.

Independent Variable		B	SE	Wald	*p*	OR	95% CI	
							LLCI	ULCI
Sample collection area	Areas other than Seoul	0.97	0.60	2.65	0.10	2.64	0.82	8.51
Age range	20–30 s	0.54	0.71	0.58	0.45	1.71	0.430	6.84
Smoking	yes	−0.08	1.24	0.004	0.95	0.93	0.08	10.45
Childbirth	2 times	0.78	1.16	0.45	0.50	2.18	0.22	21.28
	3 or more times	0.79	1.17	046	0.50	2.21	0.22	22.06

Reference group (category): Sample collection area; Seoul, Age range; 40 s, Smoking; no, Childbirth; 1 time. B, estimates; CI, confidence interval; LLCI: lower level of the 95% confidence interval; OR, odds ratio; SE, standard error; ULCI: upper level of the 95% confidence interval; Areas other than Seoul: Gyeonggi-do, Incheon, and Chungcheong-do.

**Table 4:** List of hyper-methylated and downregulated mRNAs involved in the nervous system and related diseases.

Group	Genes	Hyper-Methylated (LogFC/*p*)	Downregulated (LogFC/FDR)	Functional Characteristics	Reference
Maternal group	*MAGI2*	1.49/0.02	−1.69/2.02 × 10^−5^	Underdevelopment of nerve dendrites and loss of synapses in nerve cells.	[24]
	*ST18*	1.45/0.01	−1.60/1.55 × 10^−4^	Knockout reduces axonal outgrowth, synaptic density, and punctate size.	[25]
	*SLIT3*	1.87/5.78 × 10^−4^	−1.55/2.63 × 10^−3^	Participate in lipopolysaccharide-induced inflammatory response, which may contribute to the pathogenesis of Parkinson’s disease.	[26]
	*PTPRD*	1.14/0.02	−1.41/0.02	Dendrite branching, length and thickness are reduced.	[27]
	*WNK2*	1.96/1.00 × 10^−3^	−1.35/1.50 × 10^−3^	Significant reduction in gliomas and meningiomas due to hyper-methylation.	[28]
	*ALDH1A2*	1.03/0.02	−1.30/8.91 × 10^−4^	Knockdown causes neurite degeneration in motor neurons.	[29]
	*COL15A1*	1.45/0.03	−1.28/5.35 × 10^−3^	Deficient mice suffer from motor impairment.	[30]
	*FERMT2*	1.00/0.04	−1.10/0.02	Downregulated expression reduces synaptic connectivity.	[31]
	*DSCAML1*	1.51/0.01	−1.10/0.02	Interferes with axonal growth in cultured neurons.	[32]
Infant group	*RYR2*	1.40/0.01	−1.21/0.04	Loss of RyR2 impairs neuronal activity-dependent remodeling of dendrites.	[33]

**Table 5:** List of hypo-methylated and upregulated mRNAs involved in the nervous system and related diseases.

Group	Genes	Hypo-Methylated (LogFC/*p*)	Upregulated (LogFC/FDR)	Functional Characteristics	Reference
Maternal group	*KL*	−1.23/0.02	1.37/5.88 × 10^−5^	Decreases long-term potentiation at CA1 synapses.	[34]
	*SERPINI2*	−1.08/0.04	1.41/7.15 × 10^−5^	Over-expressed in Alzheimer’s disease (AD).	[35]
	*GINS1*	−0.89/0.04	1.13/1.16 × 10^−3^	Over-expressed in glioblastoma multiforme (GBM).	[36]
	*SKA3*	−0.66/0.05	1.13/2.09 × 10^−3^	Over-expressed in GBM.	[37]
	*IQCK*	−1.11/0.01	1.07/4.24 × 10^−3^	Over-expressed in astrocytes, neurons, and oligodendrocytes in AD brain.	[38]
	*PLOD2*	−1.07/0.04	1.02/8.86 × 10^−3^	Upregulated in glioma.	[39]
	*PTPRG*	−1.10/0.01	1.01/8.16 × 10^−3^	Over-expressed in AD.	[40]
	*VIPR2*	−1.36/0.02	1.01/7.08 × 10^−3^	Hypo-methylated in GBM.	[41]
	*TPRG1*	−1.42/1.91 × 10^−3^	1.01/0.01	Over-expressed in AD (Women-specific).	[42]
	*DLGAP5*	−1.22/0.01	0.96/0.01	Over-expression in Gliomas.	[43]
	*CYP4F3*	−0.87/0.03	0.95/0.01	Over-expressed in AD.	[44]
	*PLEKHA4*	−1.12/0.04	0.91/0.02	Over-expressed in GBM.	[45]
	*GGT5*	−1.08/0.01	0.95/0.03	Over-expressed in GBM.	[46]
	*CEP55*	−0.74/0.03	0.87/0.05	Over-expressed promotes glioma cell invasion.	[47]
Infant group	*COL5A3*	−1.59/2.50 × 10^−4^	2.00/5.11 × 10^−11^	Upregulated in a mouse model of neuropathic pain.	[48]
	*NUDT6*	−0.95/0.01	1.59/1.11 × 10^−5^	Over-expression increases anxiety and depression-like behavior in mice.	[49]
	*ANGPTL4*	−1.91/3.11 × 10^−5^	1.36/3.19 × 10^−5^	Over-expressed in GBM, usually associated with poor prognosis.	[50]
	*SPP1*	−1.43/7.34 × 10^−4^	1.07/6.68 × 10^−3^	Upregulated in mild cognitive impairment (MCI).	[51]
	*SPOCK1*	−1.69/4.08 × 10^−5^	1.05/0.03	Expression is significantly upregulated in recurrent GBM.	[52]
	*ADAMDEC1*	−1.13/0.03	1.04/0.04	The higher the expression, the higher the malignancy of glioma and the worse the prognosis.	[53]
	*APOE*	−1.03/0.02	1.02/0.01	Over-expressed and contributes to the pathogenesis of late-onset AD (LOAD).	[54]
	*DAAM2*	−1.48/1.07 × 10^−3^	0.99/0.03	Over-expression accelerates glioma tumor development.	[55]

FC, fold change; FDR, false discovery rate.

**Table 6:** Significantly upregulated mRNAs involved in the inflammatory response in the high-exposure group.

Group	Genes	LogFC	FDR	Functional Characteristics	Reference
Maternal group	*IL6*	2.64	7.37 × 10^−24^	Neuroinflammation and neuron degeneration.	[60]
	*CXCL10*	1.95	2.36 × 10^−12^	Upregulated in various neurodegenerative diseases.	[59]
	*TNFAIP6*	1.89	1.18 × 10^−11^	Upregulation is associated with poor prognosis in patients with glioblastoma multiforme.	[61]
	*IDO1*	1.83	2.38 × 10^−10^	Over-expressed in Alzheimer’s disease (AD).	[62]
	*FFAR3*	1.19	5.06 × 10^−4^	Upregulated in early stages of AD pathology.	[63]
	*TNIP3*	1.14	9.51 × 10^−4^	Over-expressed in Parkinson’s disease.	[64]
	*CHI3L1*	1.09	1.84 × 10^−3^	Expressed during nerve degeneration.	[65]
	*ORM1*	1.09	1.92 × 10^−3^	Upregulated in patients with sporadic amyotrophic lateral sclerosis (sALS).	[66]
	*PLA2G2D*	1.17	4.50 × 10^−3^	Over-expressed in Down’s syndrome (DS).	[67]
	*IL1RN*	0.96	8.69 × 10^−3^	Upregulated in patients with sALS.	[66]
	*ADORA2A*	0.91	0.02	Upregulated when synapses in neurons are damaged.	[68]
Infant group	*CXCL10*	1.13	0.04	Upregulated in various neurodegenerative diseases.	[59]
	*C3AR1*	0.91	0.04	Confirmed that over-expression correlates with cognitive decline in patients with AD.	[69]

FC, fold change; FDR, false discovery rate.

## Data Availability

The data presented in this study are available on request from the corresponding author due to (specify the reason for the restriction).
